# Mechanistic aspects of the cytotoxic activity of glufosfamide, a new tumour therapeutic agent

**DOI:** 10.1054/bjoc.1999.0974

**Published:** 2000-01-18

**Authors:** H Seker, B Bertram, A Bürkle, B Kaina, J Pohl, H Koepsell, M Wießler

**Affiliations:** Division of Molecular ToxicologyDivision of Tumor Virology, German Cancer Research Center, Heidelberg, D-69120, Germany; Division of Applied Toxicology, University of Mainz, Germany; ASTA Medica, Frankfurt/M, Germany; Institute of Anatomy, University of Würzburg, Germany

**Keywords:** β- D -glucosyl-ifosfamide mustard, tumour therapy, poly(ADP-ribose) polymerase, DNA repair deficiency

## Abstract

β- D -Glucosyl-ifosfamide mustard (D 19575, glc-IPM, INN = glufosfamide) is a new agent for cancer chemotherapy. Its mode of action, which is only partly understood, was investigated at the DNA level. In the breast carcinoma cell line MCF7 glufosfamide inhibited both the synthesis of DNA and protein in a dose-dependent manner, as shown by the decreased incorporation of [^3^H-methyl]-thymidine into DNA and [^14^C]-methionine into protein of these cells. Treatment of MCF7 cells with 50 μM glufosfamide was sufficient to trigger poly(ADP-ribose) polymerase (PARP) activation, as revealed by immunofluorescence analysis. Both CHO-9 cells, which are O^6^-methylguanine-DNA methyltransferase (MGMT)-deficient, and an isogenic derivative, which has a high level of MGMT, showed the same cytotoxic response to β- D -glc-IPM, indicating that the O^6^position of guanine is not the critical target for cytotoxicity. By contrast, a sharp decrease in survival of cross-link repair deficient CL-V5 B cells was observed already at concentrations of 0.1 m Mβ- D -glc-IPM, whereas the wild-type V79 cells showed a 90% reduction in survival only after treatment with 0.5 m M of this compound. The therapeutically inactive β- L -enantiomer of glufosfamide also showed genotoxic effects in the same assays but at much higher doses. This was probably due to small amounts of ifosfamide mustard formed under the conditions of incubation. The results indicate that the DNA crosslinks are the most critical cytotoxic lesions induced by β- D -glc-IPM. © 2000 Cancer Research Campaign


British Journal of Cancer (2000) **82**(3), 629–634
© 2000 Cancer Research Campaign
DOI: 10.1054/ bjoc.1999.0974, available online at http://www.idealibrary.com on IDEAL®

### Mechanistic aspects of the cytotoxic activity of glufosfamide, a new tumour therapeutic agent

**H Seker[1], B Bertram[1], A Bürkle[2], B Kaina[3], J Pohl[4], H Koepsell[5] and M Wießler[1]**

[1]Division of Molecular Toxicology, [2]Division of Tumor Virology, German Cancer Research Center, D-69120 Heidelberg, Germany; [3]Division of Applied Toxicology, University of Mainz, Germany; [4]ASTA Medica, Frankfurt/M, Germany; [5]Institute of Anatomy, University of Würzburg, Germany

**Summary** β-D-Glucosyl-ifosfamide mustard (D 19575, glc-IPM, INN = glufosfamide) is a new agent for cancer chemotherapy. Its mode of action, which is only partly understood, was investigated at the DNA level. In the breast carcinoma cell line MCF7 glufosfamide inhibited both the synthesis of DNA and protein in a dose-dependent manner, as shown by the decreased incorporation of [$^{3}$H-methyl]-thymidine into DNA and [$^{14}$C]-methionine into protein of these cells. Treatment of MCF7 cells with 50 μM glufosfamide was sufficient to trigger poly(ADP-ribose) polymerase (PARP) activation, as revealed by immunofluorescence analysis. Both CHO-9 cells, which are $O^6$-methylguanine-DNA methyltransferase (MGMT)-deficient, and an isogenic derivative, which has a high level of MGMT, showed the same cytotoxic response to β-D-glc-IPM, indicating that the $O^6$ position of guanine is not the critical target for cytotoxicity. By contrast, a sharp decrease in survival of cross-link repair deficient CL-V5 B cells was observed already at concentrations of 0.1 mM β-D-glc-IPM, whereas the wild-type V79 cells showed a 90% reduction in survival only after treatment with 0.5 mM of this compound. The therapeutically inactive β-L-enantiomer of glufosfamide also showed genotoxic effects in the same assays but at much higher doses. This was probably due to small amounts of ifosfamide mustard formed under the conditions of incubation. The results indicate that the DNA crosslinks are the most critical cytotoxic lesions induced by β-D-glc-IPM. © 2000 Cancer Research Campaign

**Keywords:** β-D-glucosyl-ifosfamide mustard; tumour therapy; poly(ADP-ribose) polymerase; DNA repair deficiency

Most of the clinically used cytostatic drugs have serious side-effects. There is much hope that gene therapy will make tumour therapy more specific, but at present gene therapy is far from being applicable to larger numbers of patients. Therefore, there is an urgent and persistent need for new drugs in cancer therapy. Several attempts have been made to minimize the side-effects of tumour therapeutics. For example, the urotoxic effects of ifosfamide are reduced by co-administration of 2-mercapto-ethane-sulphonate (Mesna) which deactivates acrolein, a metabolite of ifosfamide in the bladder, but Mesna itself can also lead to unwanted effects (Shaw and Graham, 1987). In view of the growing importance of pharmacokinetics in drug design and early clinical trials, new drugs for cancer chemotherapy should have low molecular weight to guarantee a clear pharmacokinetic behaviour (Workman, 1997). The glucose-coupled ifosfamide mustard D19575 (glc-IPM, INN = glufosfamide) (Pohl et al, 1995) is a compound which meets these requirements.

The major toxic effects of ifosfamide are attributed to its metabolites, especially ifosfamide mustard (IPM) and the urotoxic acrolein. In glufosfamide, IPM is coupled to C1 of glucose in an ester-like bond. Because of its glucose moiety the compound is preferably taken up by cancer cells rather than by normal cells. Inside the cell it is cleaved by glucosidases, thus liberating the cytostatic IPM (Seker et al, 1996; Seker, manuscript in preparation).

*Received 19 November 1998*
*Revised 2 August 1999*
*Accepted 5 August 1999*

*Correspondence to:* B Bertram

Glufosfamide shows a lower myelotoxicity and a higher anti-tumour activity than ifosfamide, as demonstrated with cultured cells and with human tumours grown in immunodeficient mice (Pohl et al, 1995; Stüben et al, 1996; Fiebig, personal communication). In July 1996, glufosfamide entered a clinical phase I trial and will enter phase II in 1999. Its mode of action is still under investigation.

Whereas whole-body autoradiography of the $^{14}$C-labelled compounds did not reveal considerable differences in the distribution of the β-D- and the β-L-isomers in rats, radioactivity appeared in brain and tumour only after the administration of β-D-glc-IPM, strongly suggesting that β-D-glc-IPM is able to cross the blood–brain barrier (Stüben et al, 1996; Schaper, manuscript in preparation). Since β-D-glc-IPM is a hydrophilic molecule, it is assumed that this is due to an active transport mechanism, and it has been hypothesized that a specific protein is responsible for this translocation. Recently, it has been reported that a sodium-coupled transporter (SAAT1 or SGLT3) is indeed responsible for the transport of β-D-glc-IPM into the cell (Veyhl et al, 1998). This is an example for drug targeting by employing a plasma membrane transporter.

The aim of the present work was to study the biological consequences of β-D-glc-IPM uptake by cells. Following up a previous study (Schaper, manuscript in preparation) we measured the effect of this new cytostatic drug on DNA and protein synthesis as well as stimulation of PARP activity. Inside the cell, glufosfamide is split mainly by glucosidases into glucose and ifosfamide mustard, but also non-enzymatic hydrolysis contributes to its decay. Since ifosfamide mustard also arises in the metabolism of ifosfamide, we investigated whether glufosfamide behaves like ifosfamide in the reaction with DNA. Therefore, we compared the response of cells deficient in $O^6$-methylguanine-DNA methyltransferase (MGMT) as well as a DNA cross-link repair deficient strain with the corresponding wild-type cells as to β-D-glc-IPM induced cell killing (Kaina et al, 1991).

### MATERIALS AND METHODS

### Chemicals

β-D-Glc-IPM was synthesized in the Chemical Research Laboratories of ASTA Medica AG, Frankfurt, Germany, according to the method described by Dickes (1988). β-L-Glc-IPM and β-D-$^{14}$C-Glc-IPM were synthesized according to a method developed by Wießler (Veyhl et al, 1998). $^{14}$C-label was introduced in the β-chloroethylamine side chain of the compounds; the specific radioactivity was 10 mCi $mmol^{-1}$. The α-/β-anomers were separated by recrystallization and column chromatography. Purity of the substances was > 99%. The absence of isophosphoramide mustard was ascertained by high-performance liquid chromatography (HPLC) and then liquid chromatography (TLC). L-[$^{14}$C]-methionine, and [$^3$H-methyl]-thymidine were purchased from Amersham (Buckinghamshire, UK).

### Cells

The generation of strains CHO-9-neo and TK47-AT17-C3 was described previously (Kaina et al, 1991). CHO-9-neo was derived from transfection of CHO-9-neo with the neo gene only; it is used as a MGMT-deficient control. The strain TK47-AT17-C3, which was transfected with neo plus human MGMT cDNA, expresses MGMT to a high level (720 fmol $mg^{-1}$ protein).

The breast carcinoma cell line MCF7 was obtained from the Tumorbank of the German Cancer Research Center, Heidelberg. The cells were maintained in RPMI-1640 medium supplemented with 10% fetal calf serum (Pan Systems, Nürnberg, Germany) and L-glutamine at 37°C and 5% carbon dioxide in 25 ml flasks until 100% confluency.

The mutant CL-V5B was derived from V79 Chinese hamster cells (here designated as V79 wild-type (wt)). The line was originally characterized as mitomycin C hypersensitive and is defective in DNA cross-link repair (Tellemann et al, 1995). Cells were kindly provided by Dr M Zdienicka, Leiden, The Netherlands. They were grown in Dulbecco's F12 medium containing 10% fetal calf serum.

### Influence of β-D- or β-L-glc-IPM on cell-proliferation

One times $10^5$ MCF7 cells were seeded into Petri dishes (3.5 cm, 4.5 ml medium) and cultivated for 72 h at 37°C. Then, cells were incubated for 24 h with β-D- or β-L-glc-IPM dissolved in standard medium (2.5 μM, 5 μM, 10 μM or 25 μM). All assays were carried out in duplicate. Untreated cells were used as control. After 24 h, the medium containing β-D-glc-IPM was removed, and cells were washed with phosphate-buffered saline (PBS) and incubated at 37°C with 2 μCi [$^3$H-methyl]-thymidine for 120 min. Cells were detached from the culture dishes with 0.25% EDTA, transferred into 15 ml Falcon tubes and washed three times with PBS, 0.1% bovine serum albumin (BSA), at 4°C. Cells were precipitated by the addition of 5 ml trichloroacetic acid (TCA) (10%) and left on ice for 20 min. Precipitates were collected by centrifugation at 770 ***g***, 4°C for 10 min and solubilized with 200 μl of formic acid. Radioactivity was measured in a Tricarb Liquid Scintillation Analyzer (Packard Instruments Groningen, The Netherlands).

### Influence of β-D- or β-L-glc-IPM on protein synthesis

One times $10^5$ MCF7 cells were seeded into Petri dishes (4.5 mL medium) and cultivated for 120 h at 37°C. Further steps were the same as described above for the proliferation assay. L-[methyl $^{14}$C]-methionine (2 μCi $ml^{-1}$) was used as radiolabelled compound. All assays were carried out in duplicate. Untreated cells were used as control.

### Influence of β-D- or β-L-glc-IPM on induction of PARP activity

For the detection of poly(ADP)-ribose synthesized in MCF7 cells as a consequence of β-D- or β-L-glc-IPM treatment, cells grown on cover-slips (60% confluence) were incubated with different concentrations of glc-IPM for different time intervals. Thereafter cells were washed with PBS and fixed with 10% ice-cold TCA. Poly(ADP-ribose)-specific immunofluorescence was performed as described (Bürkle et al, 1993). Cells treated with γ-rays (10 min at 8.4 Gy $min^{-1}$) were used as positive controls.

### Influence of β-D- or β-L-glc-IPM on cytotoxicity in repair-deficient strains

Cells were routinely grown in Dulbecco's modified Eagle's medium (DMEM) F12 medium (1:1) supplemented with 5% of inactivated fetal calf serum and G418 (1.5 mg/ml). G418 was omitted during the experiments. For determination of survival of CL-V5B and V79 wt cells as well as CHO-9 and TK47-AT17-C3 cells upon treatment with glufosfamide, colony formation was assayed. Cells were seeded (500–6000 cells per 5-cm dish) and treated 6 h later by adding appropriate amounts of the stock solution to the culture medium. If not stated otherwise, cells were exposed to glufosfamide for 60 min. Thereafter they were rinsed once with medium and fresh medium was added. After 6–8 days, plates were rinsed with PBS and colonies were fixed with methanol and stained with Giemsa-crystal-violet. Relative cell survival was expressed as colonies per treatment level/colonies on the control plates. Data are means of at least three independent experiments.

### RESULTS

In previous experiments, genotoxic activity of β-D-glc-IPM was shown by using the comet assay (Schaper et al, manuscript in preparation). Here we continued this study by investigating the effects of glufosfamide on proliferation and on cellular DNA monitored by the DNA strand-break-dependent formation of poly(ADP-ribose). Furthermore we studied β-D-glc-IPM cytotoxicity in repair-deficient cells in order to investigate the relevance of possible $O^6$-alkylating or cross-linking effects of glufosfamide.

Figure 1A shows that β-D-glc-IPM induced a concentration-dependent decrease in proliferation as measured by the incorporation of [$^3$H-methyl]-thymidine into DNA of MCF7 cells. Starting with a 10% decrease after addition of 2.5 μM β-D-glc-IPM to the cells, the inhibition of DNA synthesis was 50% at 25 μM. The

 © 2000 Cancer Research Campaign

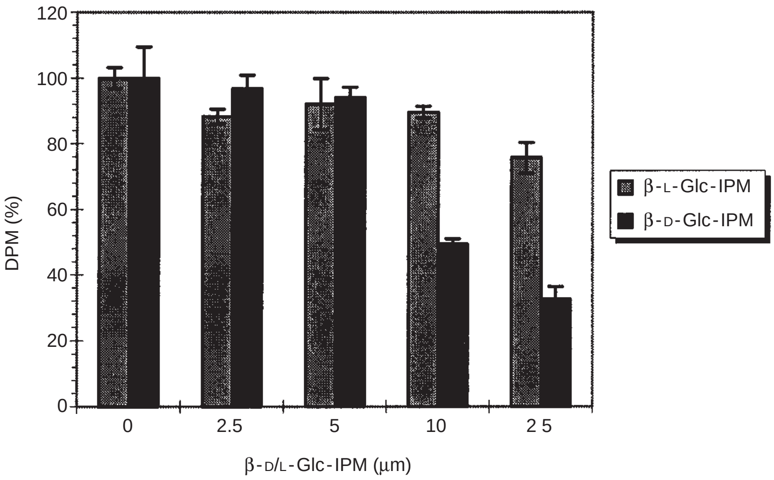

**Figure 1** Effects of various concentrations of β-D-glc-IPM and β-L-glc-IPM on DNA (**A**) and protein (**B**) biosynthesis in MCF7 cells. Radioactivity (DPM) measured in the control cultures has been set as 100%

effect of β-L-glc-IPM was much less pronounced, showing a 25% inhibition of DNA synthesis only at the highest concentration (25 μM) of this isomer.

Figure 1B shows that 10 μM and 25 μM β-D-glc-IPM lowered the protein synthesis in MCF7 cells, measured by $^{14}$C-methionine incorporation into protein of these cells by 50% and about 70%, respectively. The influence of β-L-glc-IPM was again less pronounced, showing a 25% inhibition of protein synthesis only at a concentration of 25 μM.

Poly(ADP-ribosyl)ation is a post-translational modification of nuclear proteins which is triggered by DNA strand-breaks and therefore can serve as a marker for genotoxic effects (Figure 2 A–H). The method developed by Bürkle et al (1993) is based on the fact that DNA strand-breaks, as induced directly by ionizing radiation or arising in the course of DNA base excision repair, induce activation of poly(ADP-ribose) polymerase. The treatment of MCF7 cells with γ-rays and subsequent immunofluorescence assay of poly(ADP-ribose) formation induced fluorescence signals in a dose-dependent manner, proving the reliability of this test system in MCF7 cells and the inducibility of poly(ADP-ribosyl)ation. Figure 2 h shows the effect of a 10-min irradiation with 8.4 Gy min$^{-1}$. The effects of an incubation of MCF7 cells with 50 μM, 250 μM and 750 μM glufosfamide for 24 h are shown in Figure 2 B,C,D. It is evident that PARP activity was triggered by β-D-glc-IPM at concentrations as low as 50 μM. The β-L-isomer led also to an induction of PARP-activity but only at a concentration of 750 μM (Figure 2F).

Survival of MGMT-deficient and MGMT-proficient CHO cells (strains CHO-9-neo and Tk47-AT17-C3 respectively) upon treatment with glufosfamide is shown in Figure 3A. There was no difference in survival between both strains indicating that $O^6$-alkylguanine, which is subject to repair by MGMT, is not formed at significant amounts after treatment with the drug, or alternatively, that this lesion does not persist long enough, perhaps due to the rapid conversion of the monoadduct into a DNA cross-link. On the other hand, the cross-link repair-deficient cell line CL-V5B was clearly hypersensitive to glufosfamide as compared to V79 wt, which is indicative of cross-link formation by the agent. It should be noted that CHO-9 cells were more resistant than V79 wt. The reason for this strain difference is unknown, but it is tempting to speculate that either cross-link repair is more efficient, or the uptake by glucose transporter occurs less efficiently in CHO than in V79 cells. A further explanation could be that the content of β-glucosidases in CHO cells necessary for metabolic activation of glufosfamide is possibly lower than that of V79 cells.

### DISCUSSION

The molecular basis for the neoplastic behaviour of advanced tumours is largely undefined (Monks et al, 1997). Notably, the target for drug intervention is unclear. Here we concentrated on effects of the cytostatic compound glufosfamide on DNA in MCF7 cells concerning proliferation and repair. Toxic effects of glufosfamide on DNA and on protein synthesis in MCF7 cells, indicative of a genotoxic activity of this compound, were observed already at low doses. The effects on DNA became apparent between 2 and 5 μM, shown by an impaired incorporation of [$^3$H-methyl]-thymidine into DNA of these cells. The L-isomer exerted similar effects but at higher concentrations. We suppose that this effect is due to partial hydrolysis of β-L-glc-IPM liberating the IPM-moiety, because cells were incubated for 24 h after which time hydrolysis has already begun. The inhibition of DNA and protein syntheses is probably due to alkylating properties of glufosfamide. IPM, which arises in the metabolism of glufosfamide, is a powerful alkylating agent (Hemminki, 1986) and may react with cellular components involved in protein synthesis like ribosomes, ribosomal RNA or proteins involved in translation processes (Monks et al, 1997), thus leading to an impairment thereof.

One of the first responses of eukaryotic cells to some types of DNA damage is the covalent modification of nuclear proteins with poly(ADP-ribose). Poly(ADP-ribosyl)ation is catalysed by the nuclear enzyme poly(ADP-ribose) polymerase (PARP; EC 2.4.2.30) which utilizes $NAD^+$ as substrate (for review see: DeMurcia and Ménissier-de Murcia, 1994; Lindahl et al, 1995; Oci et al, 1997). The two zinc fingers in the aminoterminal DNA-binding domain of PARP mediate the recognition of single- or

© 2000 Cancer Research Campaign 

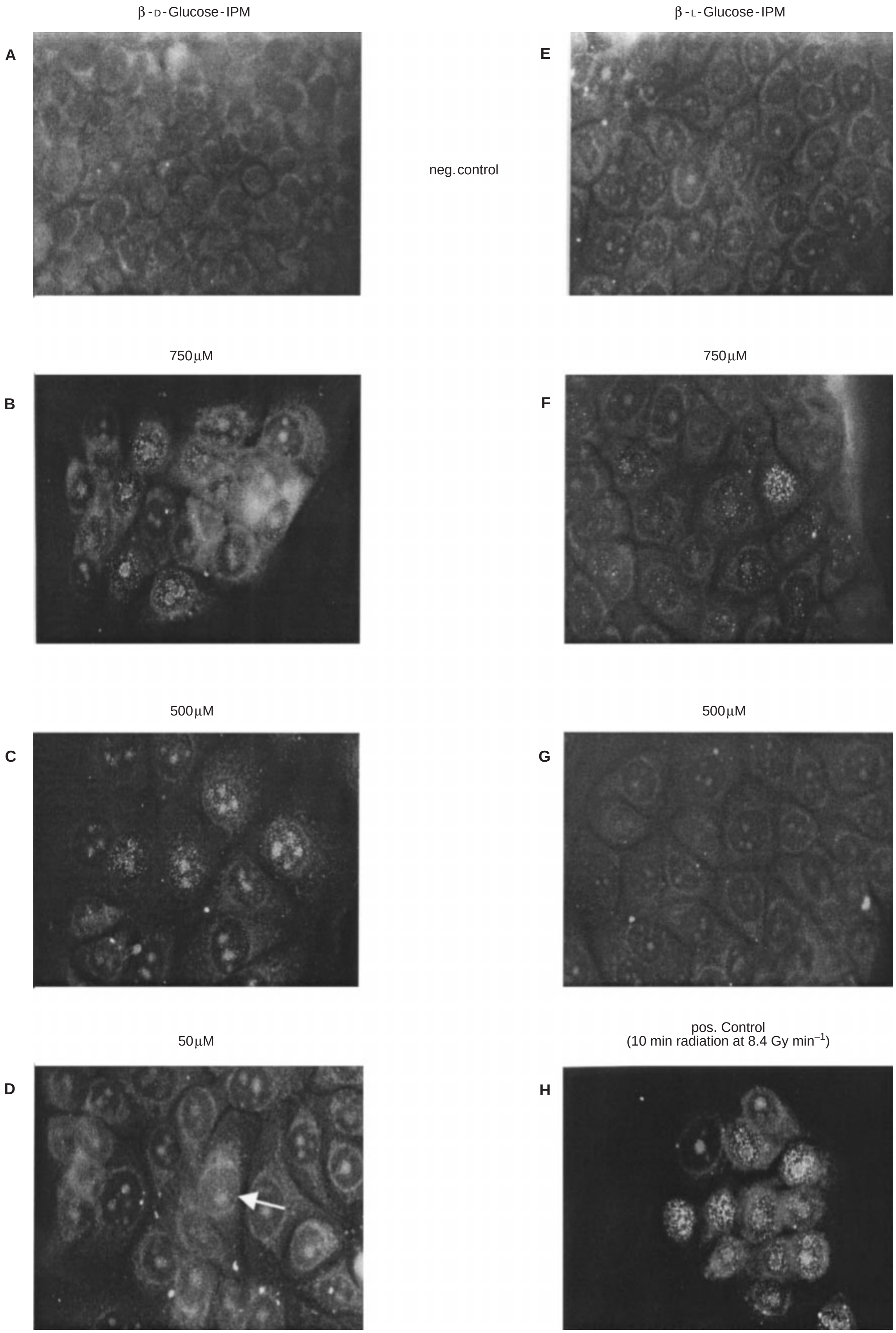

**Figure 2** Immunodetection of poly(ADP-ribose) induced by various concentrations of β-D-glc-IPM in MCF7 cells. Cells were treated for 24 h. β-L-Glc-IPM which has no cytostatic activity has been used as control substance. (**A**) β-D-glc-IPM 0 µM; (**B**), 750 µM; (**C**), 500 µM; (**D**), 50 µM; (**E**), β-L-glc-IPM 0 µM; (**F**), 750 µM; (**G**), 500 µM; (**H**), positive control (10 min at 8.4 Gy $min^{-1}$)

*© 2000 Cancer Research Campaign*

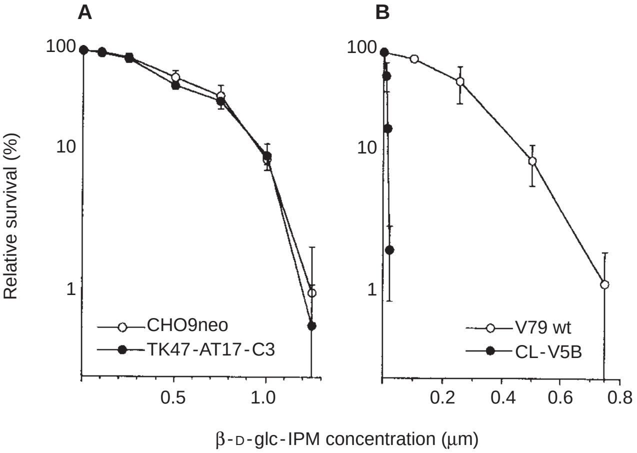

**Figure 3** Effect of β-D-glc-IPM on survival of repair-proficient and repair-deficient Chinese hamster cell lines. (**A**) Comparison of the cytotoxic response of CHO-9-neo and TK-47-AT17-C3 cells not expressing and expressing MGMT respectively. (**B**) Comparison of cytotoxic response of V79 wild-type (wt) and DNA cross-link repair-deficient line CL-V5B. Survival was measured by determination of colony forming ability. Data are the mean of at least 3 separate experiments ± s.d.

double-strand-breaks in DNA, thus triggering the activation of the catalytic centre in the carboxyterminal $NAD^+$-binding domain. As a consequence, exposure of cells to certain chemical or physical DNA-damaging agents (including reactive oxygen species, alkylating agents, and γ-radiation) induces a dose-dependent stimulation of cellular poly(ADP-ribose) synthesis.

While the biological functions of PARP have not yet been fully eludicated at the molecular level, cellular poly(ADP-ribose) formation may be used as a marker for the infliction of at least some types of DNA damage, and this can be conveniently assessed in situ by immunofluorescence (Bürkle et al, 1993), using a monoclonal antibody directed against poly(ADP-ribose) (Kawamitsu et al, 1984). Together with previous results on genotoxic effects of glufosfamide as revealed by the comet assay (Schaper et al, manuscript in preparation), the data presented here strongly suggest DNA alkylating properties of glufosfamide. However, in the comet assay the concentration needed to induce detectable DNA damage was very high (10 mM). By contrast, in the present work we could demonstrate that PARP activity, as detected by an immunofluorescence assay (Bürkle et al, 1993), was induced already at a much lower concentration of glufosfamide (50 μM). The question of whether this DNA-damage was mainly due to $O^6$-alkylation processes or to cross-links produced by glufosfamide was addressed by using repair-deficient cell strains. It became apparent that cross-linking was much more toxic than $O^6$-alkylation. The concentrations of glufosfamide needed to produce DNA damage either in the PARP or in the cross-linking assay were in the μM range. In contrast, in the comet assay the appropriate concentrations were in the mM range. We suppose that this discrepancy is a result of the strong cross-linking effects of the metabolite ifosfamide mustard, preventing the DNA-pieces from forming the characteristic comet at concentrations which show activation of PARP. Similar results regarding cross-linking agents investigated in the comet assay have been described (Pfuhler and Wolf, 1996). Experiments are underway to investigate whether suppression of PARP by appropriate inhibitors may increase the therapeutic potency of glufosfamide. The L-isomer led also to an activation of repair mechanisms but the concentrations needed were more than ten times higher than with the β-D-isomer. The reason is again seen in the formation of IPM which is hydrolytically formed after 24 h of incubation. Results from our comparison of repair-deficient and -proficient strains showed that glufosfamide obviously did not induce DNA $O^6$-alkylation which can be repaired by MGMT, but led to cross-link formation. Together with other results reported here, this shows a certain similarity of glufosfamide to ifosfamide suggesting that its cytotoxic effects are mainly due to IPM, the metabolite of both compounds. Yet the toxicity of glufosfamide in white blood cells, colony forming units and spleen colony forming units is considerably lower as compared to ifosfamide (4). Another exciting difference between ifosfamide and glufosfamide was demonstrated by Volm and colleagues who were able to provoke resistance of cells upon continuous drug treatment against ifosfamide but not against glufosfamide in $SKOV_3$ cells (Volm, personal communication).

A necessary requirement for the therapeutic efficacy of saccharide-conjugates is their activation in the target cells. We suppose that the conjugates have to be split by glucosidases to liberate the therapeutic moiety. In a forthcoming paper we will report on the correlation between the content of β-glucosidases in different cell lines and their sensitivity against glufosfamide.

In conclusion, we have shown that the cytostatic compound glufosfamide impaired protein and DNA synthesis in MCF7 cells and that it triggered the activation of PARP, an enzyme specifically sensing DNA strand-breaks, at concentrations of 5 μM and 50 μM respectively. The major cytotoxic lesions are probably due to cross-linking effects rather than monofunctional alkylations exerted by glufosfamide, as shown by comparison of isogenic cell-lines deficient and proficient of cross-link repair. In view of the minute amounts of glufosfamide taken up by the cells (Schaper et al, manuscript in preparation), it is surprising that very low concentrations of the drug can lead to the observed striking effects at the DNA level. Further investigations on this issue are in progress.

### ACKNOWLEDGEMENTS

Hasan Seker had a scholarship from the Schlosser Stiftung. We are grateful to ASTA Medica for the generous gift of glufosfamide and to Dr M Zdienicka (Leyden) for providing us with CL-V5B cells.

### REFERENCES

Bürkle A, Chen G, Küpper J-H, Grube K and Zeller WJ (1993) Increased poly(ADP-ribosyl)ation in intact cells by cisplatin treatment. *Carcinogenesis* **14**: 559–561

De Murcia G and Ménissier-de Murcia J (1994) Poly(ADP-ribose) polymerase: a molecular nick sensor. *Trends Biochem Sci* **19**: 172–176

Dickes M (1988) Synthese und Untersuchungen zur Stabilität und biologischen Aktivität O-glykosidisch gekoppelter 2-Chlorethyl-phosphorsäurediamide – einer neuen Klasse antineoplastisch wirksamer Alkylantien. PhD thesis, University of Heidelberg

Hemminki K, (1986) *Reactions of Nitrogen Mustards with DNA*. IARC Scientific Publication **78**, pp. 55–70. IARC: Lyon

Kaina B, Fritz G, Mitra S and Coquerelle T (1991) Transfection and expression of human $O^6$-methylguanine-DNA methyltransferase (MGMT) CDNA in Chinese hamster cells: the role of MGMT in protection against the genotoxic effects of alkylating agents. *Carcinogenesis* **12**: 1857–1867

Kawamitsu H, Hoshino H, Okada H, Miwa M and Sugimura T (1984) Monoclonal antibodies to poly(adenosine diphosphate ribose) recognize different structures. *Biochemistry* **23**: 3771–3777

© 2000 Cancer Research Campaign 

Lindahl T, Satoh MS, Poirier G G and Klungland A (1995) Post-translational modification of poly(ADP-ribose) polymerase induced by DNA strand breaks. *Trends Biochem Sci* **20**: 405–411

Monks A, Scuderio DA, Johnsson DS, Paull KD and Sausville EA (1997) The NCI-anti-cancer screen: a smart screen to identify effectors of novel targets. *Anti Cancer Drug Design* **12**: 533–541

Oei SL, Griesenbeck J and Schweiger M (1997) The role of poly ADP-ribosylation. *Rev Physiol Biochem Pharmacol* **131**: 4135–4137

Pfuhler S and Wolf HU (1996) Detection of DNA-crosslinking agents with the alkaline comet assay. *Environment Mol Mutag* **27**: 196–201

Pohl J, Bertram B, Hilgard P, Nowrousian MR, Stüben J and Wießler M (1995) D-19575 – a sugar-linked isophosphoramide mustard derivative exploiting transmembrane glucose transport. *Cancer Chemothe. Pharmacol* **35**: 364–370

Seker H, Bertram B and Wieβler M (1996) *Possible role of the cytosolic β-glucosidase in the metabolism of saccharide-coupled platinum and ifosfamide mustard in tumor cells*. In '10th Mediterranean Congress of Chemotherapy' Berkarda, B. (ed.), pp 381–385, Monduzzi Editore

Shaw IC, Graham MI (1987) Mesna – a short review. *Cancer Treatment Rev* **14**: 67–86

Stüben J, Port R, Bertram B, Bollow U, Hull E, Schaper M, Pohl J and Wießler M (1996) Pharmacokinetics and whole body distribution of the new chemotherapeutic agent β-D-glucosylifosfamide mustard (D 19575) and its effects on the incorporation of [$^3$H-methyl]-thymidine in various tissues of the rat. *Cancer Chemother Pharmacol* **38**: 355–365

Tellemann P, Overkamp WJI, van Wessel N, Studzian K, Wetselaar L, Natarajan AT and Zdienicka MZ (1995) A new complementation group of mitomycin C-hypersensitive Chinese hamster cell mutants that closely resembles the phenotype of Fanconi anemia cells. *Cancer Res* **55**: 3412–3416

Veyhl M, Wagner K, Volk C, Gorboulev V, Baumgarten K, Weber WM, Schaper M, Bertram B, Wießler M and Koepsell H (1998) Transport of the new chemotherapeutic agent β-D-glucosyl-isophosphoramide mustard (D-19575) into tumor cells is mediated by the $Na^+$-D-glucose cotransporter SAAT1. *Proc Natl Acad Sci USA* **95**: 2914–2919

Workman P (1997) Towards intelligent anticancer drug screening in the post-genome era? *Anti-Cancer Drug Design* **12**: 525–531

 *© 2000 Cancer Research Campaign*
